# Systematic Review and Meta-Analysis of Congenital Toxoplasmosis Diagnosis: Advances and Challenges

**DOI:** 10.1155/2024/1514178

**Published:** 2024-02-21

**Authors:** Priscila Silva Franco, Ana Carolina Morais Oliveira Scussel, Rafaela José Silva, Thadia Evelyn Araújo, Henrique Tomaz Gonzaga, Camila Ferreira Marcon, Joaquim Pedro Brito-de-Sousa, Angélica Lemos Debs Diniz, Marina Carvalho Paschoini, Bellisa Freitas Barbosa, Olindo Assis Martins-Filho, José Roberto Mineo, Eloisa Amália Vieira Ferro, Angelica Oliveira Gomes

**Affiliations:** ^1^Universidade Federal de Uberlândia, Avenida João Naves de Ávila 2121, Uberlândia, Santa Mônica 38408-100, MG, Brazil; ^2^Universidade Federal do Triângulo Mineiro, Rua Frei Paulino, 30, Nossa Sra. da Abadia 38025-180, Uberaba, MG, Brazil; ^3^Instituto René Rachou, Fundação Oswaldo Cruz, Avenida Augusto de Lima, 1715, Barro Preto 30190-002, Belo Horizonte, MG, Brazil

## Abstract

**Objective:**

To understand how congenital toxoplasmosis (CT) diagnosis has evolved over the years, we performed a systematic review and meta-analysis to summarize the kind of analysis that has been employed for CT diagnosis.

**Methods:**

PubMed and Lilacs databases were used in order to access the kind of analysis that has been employed for CT diagnosis in several samples. Our search combined the following combining terms: “congenital toxoplasmosis” or “gestational toxoplasmosis” and “diagnosis” and “blood,” “serum,” “amniotic fluid,” “placenta,” or “colostrum.” We extracted data on true positive, true negative, false positive, and false negative to generate pooled sensitivity, specificity, and diagnostic odds ratio (DOR). Random-effects models using MetaDTA were used for analysis.

**Results:**

Sixty-five articles were included in the study aiming for comparisons (75.4%), diagnosis performance (52.3%), diagnosis improvement (32.3%), or to distinguish acute/chronic infection phases (36.9%). Amniotic fluid (AF) and placenta were used in 36.9% and 10.8% of articles, respectively, targeting parasites and/or *T. gondii* DNA. Blood was used in 86% of articles for enzymatic assays. Colostrum was used in one article to search for antibodies. In meta-analysis, PCR in AF showed the best performance for CT diagnosis based on the highest summary sensitivity (85.1%) and specificity (99.7%) added to lower magnitude heterogeneity.

**Conclusion:**

Most of the assays being researched to diagnose CT are basically the same traditional approaches available for clinical purposes. The range in diagnostic performance and the challenges imposed by CT diagnosis indicate the need to better explore pregnancy samples in search of new possibilities for diagnostic tools. Exploring immunological markers and using bioinformatics tools and *T. gondii* recombinant antigens should address the research needed for a new generation of diagnostic tools to face these challenges.

## 1. Introduction

Congenital toxoplasmosis (CT) is a severe form of the disease caused by *Toxoplasma gondii* and occurs through the transplacental passage of tachyzoites from pregnant women to the fetus [1]. The risk of transmission depends on gestational age and clinical management for effective therapeutic intervention [2]. Infected fetuses and newborns can suffer serious consequences of infection, such as retinochoroiditis, encephalitis, intracranial calcification, hydrocephalus, and death [3].

Effective control and treatment of CT depend on accurate detection of *T. gondii* infection. The utilization of highly sensitive and specific diagnostic methods followed by treatment can prevent placental transmission, fetal infection, and sequelae to the fetus [4]. The laboratory diagnosis approaches commonly employed in CT are based on molecular, parasitological, and immunological assays such as PCR, bioassays, and immunoenzymatic assays, respectively. These methods allow detecting the parasite or antibodies using different samples such as amniotic fluid (AF) [5–23], umbilical cord blood, maternal and newborn blood [14–69], placental fragments [62–68], and colostrum [69]. There are still numerous gaps in CT diagnosis. The variability in diagnostic performance, adding to the difficulty in interpreting results to differentiate the infection stage in pregnant women, causes delays in diagnosis and treatment. Diagnostic failures are also associated with unnecessary amniocentesis or poorly designed treatment. All these conditions compromise gestational safety.

Procedures adopted to diagnose the infection, including test type/platform and antenatal period, vary according to the guidelines of each country/society. For example, in Brazil [70], CT is confirmed when a suspected case presents one of the following situations: presence of *T. gondii* DNA in AF, fetal tissue, or child body fluids; IgM or IgA and anti-*T. gondii* IgG reagent up to six months of life; serum levels of anti-*T. gondii* on the rise in at least two serial samples with a minimum interval of 3 weeks during the first 12 months of life; anti-*T. gondii* IgG persistently reactive after 12 months of age; retinochoroiditis, hydrocephalus, or cerebral calcification (or associations between the signs) with reactive IgG.

Several countries have CT surveillance programs, but robust information on the frequency of CT transmission is limited to a few countries [71], so CT is substantially underestimated worldwide [72]. Despite this, published data show that *T. gondii* is responsible for almost two-thirds of the estimated 1.9 million disability-adjusted life years (DALYs) [73], with an estimated 190,000 cases annually [74]. The incidence estimation of CT can be obtained from case report series, inferences from gestational toxoplasmosis, and testing babies at birth [71]. The disease is associated with fetal loss and neonatal death in approximately 3% of cases, [75] as well as craniocerebral/ocular sequelae [76]. Subclinical disease at birth is present in 75% of cases, with symptoms that may start many years or even decades later [74].

To provide an understanding of the evolution of CT diagnosis over the years, we present here a review of methods that are currently employed for prenatal and postnatal CT diagnosis in several samples. It emphasizes the sample type, targets, and methods applied to diagnosis at different gestational ages using biological samples from pregnant women, fetuses, and newborns. Moreover, it brings insights into possible future challenges of CT diagnosis.

## 2. Methods

### 2.1. Search Strategy, Study Selection, and Data Extraction

Our study followed the preferred reporting items for systematic reviews (PRISMA) guidelines [46]. PubMed and Lilacs citation databases were searched from 2001 to 2020, combining the terms “congenital toxoplasmosis” or “gestational toxoplasmosis” and “diagnosis” and “blood” or “serum” or “amniotic fluid” or “placenta” or “colostrum.” Only papers using human samples and written in English were included.

The articles were selected by the Rayyan program, and seven authors conducted the preliminary selection based on abstracts and paper titles. After the first selection, conflicting decisions by at least three authors were considered for a second blind analysis. Afterward, articles considered eligible by at least four authors were included in the preliminary screening for full reading. Studies with at least one of the following criteria were excluded: studies evaluating exclusively infant samples, reviews or descriptive studies, articles with no eligible data, case reports, and studies approaching multiple infections.

The following data were recorded from the selected studies: major goals, sample type, gestational age at sample collection, laboratory methods, and major results. For meta-analysis, data on molecular (PCR) and bioassay diagnosis performance were collected from articles that included these analyses and provided data about the number of samples, sensitivity, and specificity. Only studies that reported the true positive, false negative, true negative, and false positive values or that these values could be calculated were included. Investigators collected data independently. When literature data interpretation was controversial, investigators discussed it and reached a consensus. Some studies considered essential to the review that were not included in any of the research bases were added to the introduction and discussion.

### 2.2. Statistical Analysis

Venn diagram was performed using Bioinformatics and Evolutionary Genomics, available at https://bioinformatics.psb.ugent.be/webtools/Venn/. It was constructed to identify common and exclusive biological samples used in the selected studies. Meta-analysis of molecular diagnosis or bioassay in AF or placenta samples was performed using MetaDTA (version 2.0) [77, 78], available at https://crsu.shinyapps.io/dta_ma/. The diagnostic odds ratio (DOR), positive likelihood (LR+), and negative likelihood ratio (LR−) were used to determine the overall diagnostic accuracy. Sensitivity and specificity points were shown along with forest plots and SROC curves. The forest plots were edited using GraphPad Prism software. Heterogeneity and threshold effects were evaluated using the visual summary of SROC plots and random effects correlation, as described by Druce et al. [79]. All summary parameters were calculated along with the associated 95% confidence interval (CI).

## 3. Results

### 3.1. Analysis of the Included Literature

**Figure 1:**
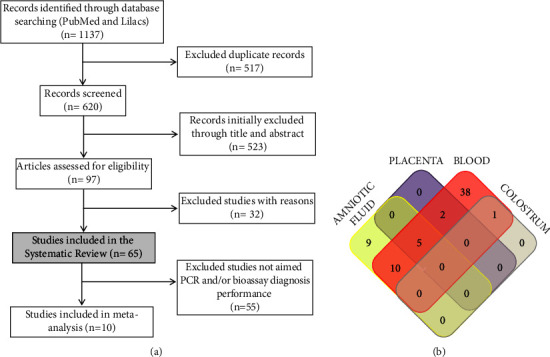
Details of search and study selection procedure. (a) The PRISMA flow diagram describing the study design process. (b) Venn diagram constructed to identify common and exclusive samples used in the included studies.

Through this systematic review, 1137 articles were found following the initial database search. In total, 517 articles were excluded from duplicate records, and 620 articles were screened based on title and abstract. From that, 523 articles were excluded, as it did not fit our filters. The remaining 97 articles were evaluated by full reading, and 32 were excluded according to the criteria outlined in the Methods. Finally, 65 articles were included in the systematic review, and of these, 10 articles aiming for diagnostic performance on molecular assays (PCR) and/or bioassay were selected for meta-analysis. Details of the search and study selection procedures were described in a PRISMA flow diagram (Figure 1(a)).

To identify common and exclusive samples used in the selected studies, a Venn diagram was constructed (Figure 1(b)). The analysis of samples used in the articles demonstrated that 9 studies were conducted employing exclusively AF, whereas 38 studies were performed exclusively with blood samples. Analyzing the articles with more than one sample, it was demonstrated that 10 articles used AF and blood, 2 articles used placenta and blood, 1 article used colostrum and blood, and 5 articles used AF, blood, and placenta simultaneously (Figure 1(b)).

### 3.2. Major Goals of the Selected Articles

The major goals explored in the 65 selected articles were analyzed (Table 1), and 34 of them aimed to analyze diagnosis performance. Fifteen articles presented diagnosis performance on molecular diagnosis and/or bioassay and 23 articles analyzed serological performance diagnosis. Twenty-two articles in this review aimed at diagnosis improvement, 9 of them using a combination of diagnosis assays to improve performance [7, 14, 15, 21, 30, 62–64, 69] and 12 employing some modification of available methodologies [6, 27, 32, 34, 39, 42–44, 46, 47, 56, 60].

Most of the articles in this review, 49 articles, aimed comparisons: performance comparisons based on time of sample collection [5, 13, 18, 22, 46], comparisons between assays [6–69], comparisons between samples [18, 20, 21, 65, 68, 69], and other comparisons [5, 22, 53].

Twenty-four articles aimed to distinguish acute/chronic phases of infection, and all of them employed blood samples [21, 24–27, 29, 31, 33, 36, 38–41, 44, 47, 51, 52, 54–57, 59–61]. Twenty-four articles presented other objectives, such as evaluating the treatment effect on CT diagnosis [5, 13, 27, 64], correlating parasite load to CT severity [6, 22], characterizing *T. gondii* strains on CT [12, 16], and others [17, 19, 23, 28, 38, 39, 42, 48–50, 58, 59, 62, 63, 67, 69].

### 3.3. Diagnostic Methods That Employed Amniotic Fluid Samples

From the careful selection, 24 articles from the total used AF as a sample (Table 2). Regarding gestational age on sample collection, 5 articles collected AF between the 14th and 26th gestational weeks (GW), 9 articles collected AF between the 14th and 41st GW, and 5 articles collected additional samples at birth. Ten articles did not provide details on the date of sample collection.

All selected articles used parasites and/or *T. gondii* DNA as targets of study. Concerning the assays performed in those studies, all selected articles performed PCR and 8 of them also performed bioassay by mouse inoculation. The B1 gene was the most commonly used gene in PCR (19/24 articles). Twelve articles (12/24 articles) used other genes such as 529-bp, RE-sequence, P30, and others. Two articles (2/24 articles) did not provide details about PCR. Most studies using bioassay did not provide information about the methodology employed.

### 3.4. Diagnostic Methods That Employed Blood Sample

Of the total, 56 articles used blood samples for CT diagnosis (Table 3). Peripheral blood samples were collected from pregnant women (M-PB) (51 articles/78.4%), cord blood by cordocentesis (P-CB) (2 articles), cord blood at the time of delivery (N-CB) (11 articles), or/and neonatal peripheral blood (N-PB) (18 articles).

When the target was examined, 53 of 56 articles analyzed antibodies against *T. gondii* by immunoassays. The performed serological methods were enzyme assays (ELISA, VIDAS, Enzygnost, Platelia, AxSYM, Cobas, EIA, WB, MEIA, and ELIFA) in 49 of 53 articles; agglutination (ISAGA, DA, HSDA, ICT, AC/HS) in 25 articles; fluorescence (IFAT, IMX, IF, FAT, ELFA, and FEIA) in 23 articles; and chemiluminescence (Architect, ECLIA, Liaison, CML, Vidia, and Advia Centaur) in 6 articles. The Sabin–Feldman dye test (SFDT) was used in 9 articles, and latex agglutination test, laser immunonephelometry, or lateral flow immunoassay (LFIA) were used in 1 article.

Analysis of parasite and/or *T. gondii* DNA in blood samples was applied in 12 of 56 articles. For parasite/DNA detection, 11 articles used PCR and 2 articles used bioassay. The B1 gene was the most commonly used in PCR.

Regarding the type of blood samples and assay employed for diagnosis, all articles with M-PB (*n* = 51) used serological methods. IgM and IgG were the most assessed molecules (IgM: 47 articles, IgG: 51, IgA: 9, and IgE: 2). IgG avidity was analyzed in 32 articles and IgG subclasses in 1 article. Enzyme assays were performed in 47 articles, agglutination assays in 19 articles, fluorescence assays in 21 articles, and chemiluminescence assays in 8 articles. Eight articles used M-PB to assess *T. gondii* DNA by PCR.

All articles with P-CB (*n* = 2) used serological methods to analyze antibodies (IgM: 2 articles, IgA: 2, IgG: 1). No article analyzed IgG subclasses or IgE. Agglutination assays were performed in all articles, and enzyme or fluorescence assays were performed in 1 article. One article used P-CB samples for bioassay, but PCR was not performed.

Nine of 11 articles used serological methods to analyze N-CB. IgM and IgG antibodies were the most assessed molecules (IgM: 9 articles, IgG: 5, IgA: 2). No article analyzed IgG avidity, IgG subclasses, or IgE. The enzyme and agglutination assays were performed in 4 articles and the fluorescence assays in 2. Four of 11 articles used N-CB to assess *T. gondii* DNA by PCR and 2 for bioassay.

Seventeen of 18 articles used serological methods to analyze N-PB. IgM and IgG were the most assessed molecules (IgM: 16 articles, IgG: 15, IgA: 5). No article has analyzed IgE. IgG avidity was analyzed in 5 articles and IgG subclasses in 1 article. Enzyme assays were performed in 14 articles, agglutination assays in 13, fluorescence assays in 8, and chemiluminescence assays in 2 articles. One article used N-PB to assess *T. gondii* DNA by PCR. No article employed N-PB to perform bioassay.

### 3.5. Diagnostic Methods That Employed Placenta and Colostrum Samples

Analyzing articles that employed samples for postnatal diagnosis, it was detected that 7 articles used placenta and 1 used colostrum for CT diagnosis (Table 4). Placentas were used to search for parasites and/or *T. gondii* DNA by PCR or bioassay. PCR and bioassay were performed in 4 studies, whereas 2 articles used exclusively PCR and 1 article used exclusively bioassay. B1 was the most commonly used gene for PCR in placentas. REP529 and RE-sequence were applied in the PCR in 2 and 1 articles, respectively. Concerning the bioassay, 2 articles employed Swiss females and 2 other articles did not report details about this methodology. Colostrum was collected up to 3 days after birth. Samples were analyzed to detect anti-*T. gondii* antibodies using ELISA and western blot immunoassays.

### 3.6. Measures of Diagnostic Performance

**Figure 2:**
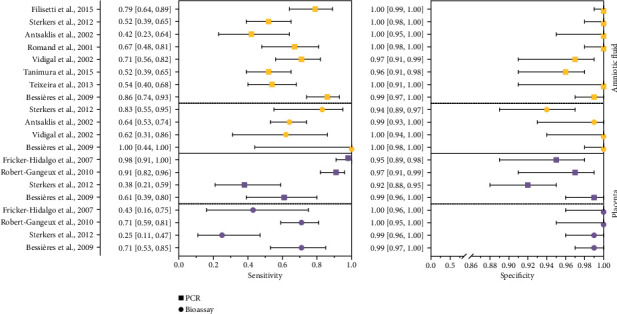
Forest plot of the sensitivity and specificity estimates and 95% confidence intervals (CI) for PCR and bioassay in amniotic fluid or placenta samples according to the single study sets. Estimates of sensitivity and specificity from each study are shown as solid yellow squares for PCR in amniotic fluid, solid yellow circle for bioassay in amniotic fluid, solid purple squares for PCR in placenta, and solid purple circle for bioassay in placenta.

From the 10 articles included in the meta-analysis, 8 used AF for PCR [5, 7, 11, 14, 19, 20, 66, 67] and 4 used AF for bioassay [7, 14, 66, 67]. Four articles used placenta for both PCR and bioassay techniques [62, 63, 66, 67]. The estimated sensitivity and specificity of PCR in AF were 85.1% (95% CI 69.5–94.4%) and 99.7% (95% CI 97.2−1.00%), respectively. The sensitivity and specificity of the bioassay in AF were 75.4% (95% CI 41.6–71.8%) and 99.3% (95% CI 93.6–99.9%), respectively. PCR in placenta had an estimated sensitivity of 58.9% (95% CI 58.5–59.3%) and a specificity of 96.3% (95% CI 96.3–96.4%). Bioassay in placenta had an estimated sensitivity of 58.6% (95% CI 47.2–69.2%) and a specificity of 99.5% (95% CI 97.9–99.9%). Paired forest plots are shown in Figure 2 and Table 5. The RE correlation for the bioassay presented values of +1 for AF and −1 for PL. For the PCR scenarios, the RE correlation values were −0.572 for PCR in AF and −0.365 in PL (Table 5).

The odds ratios determined by PCR or bioassay of AF and placenta were combined for quantitative comparison. DOR was 2018.385 (95% CI 228.652–17816.960) for PCR in AF, 189.94 (95% CI 13.45–2681.75) for bioassay in AF, 37.70 (95% CI 36.8–38.58) for PCR in placenta, and 258.86 (95% CI 69.77–960.40) for bioassay in placenta (Table 4).

PCR in AF showed higher LR+ (302.048 with 95% CI 30.916–2950.945) and lower LR− (0.150 with 95% CI: 0.069–0.325) compared to the other techniques (Table 5).

## 4. Discussion

To the best of our knowledge, the first conclusive reported case of toxoplasmosis in newborns was diagnosed based on encephalomyelitis and chorioretinitis findings in infant postmortem tissues. Mice and rabbit tissue inoculations evidenced an infection with protozoa morphologically compatible with *T. gondii*. [80] Afterward, a dye test was developed to evaluate the presence of the specific antibody [81]. Following, a description of *T. gondii* isolation from the placenta gave a new puzzle connection about congenital infection [82]. Later, a 15-year prospective study brought important information about CT diagnosis [83].

**Figure 3:**
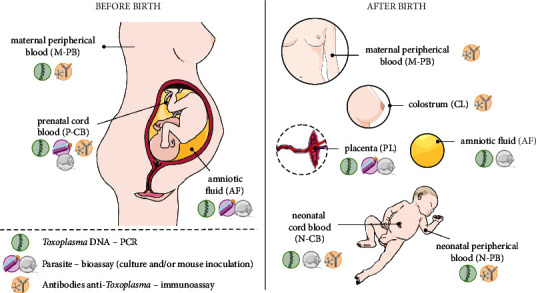
Schematic model represents the types of samples and methods used to *T. gondii* detection before and after birth. Maternal peripheral blood (M-PB), prenatal cord blood (P-CB), and amniotic fluid (AF) are the samples collected for gestational and congenital toxoplasmosis diagnosis before birth. After birth, the samples that can be collected are maternal peripheral blood (M-PB), colostrum (CL), placenta (PL), amniotic fluid (AF), neonatal cord blood (N-CB), and neonatal peripheral blood (N-PB) to confirm the congenital toxoplasmosis. The target and methods most used to detect the infection are *Toxoplasma* DNA by PCR; the parasite by bioassay (culture and/or mouse inoculation); and antibodies anti-*Toxoplasma* by immunoassay. PCR: polymerase chain reaction. Figure created using images from Servier medical art by Servier licensed under creative commons attribution 3.0 France (CC BY 3.0 FR).

The present study aimed to investigate commonly used diagnostic methods for CT and understand the accuracy of these methodologies. From these data, we seek out new diagnostic proposals that can be investigated, bringing insights into new diagnostic approaches. Our data suggested that, in the last 20 years, the samples and assays used for CT diagnosis are basically the same as those of past decades. Few studies evaluated the effectiveness of alternative samples, such as colostrum. The majority of the studies in this review used blood samples mainly for serological screening, and a few studies used more than one type of sample for diagnostic investigation. The increase in amount and time of sample collection represents a gain for CT diagnosis that has also evolved in accuracy [30]. A schematic model representing the types of samples and methods used for *T. gondii* detection before and after birth is shown in Figure 3.

Techniques used for CT diagnosis have advanced; however, many difficulties are still encountered in screening pregnant women and fetuses. Our review suggested that one of the major challenges of CT diagnosis is dating the *T. gondii* infection [30]. *T. gondii*-specific immunoglobulin (IgG and IgM) searches are often used to investigate when the infection occurred [3, 25, 30]. IgG-avidity helps determine the risk of *T. gondii* transmission at any time during pregnancy [29]. Conversely, avidity assay results classified as borderline or low can be erroneously interpreted as consistent with a recently acquired infection [25].

Many studies in this review aimed to distinguish between acute/chronic infection phases. However, few of them used new approaches, such as evaluating more options for *T. gondii* antigens for the improvement of enzyme assays [26, 33, 39, 44, 47] or employing bioinformatics tools, such as epitope prediction for CT diagnosis [56]. The use of specific molecular markers is a promising option in *T. gondii* serodiagnosis and can be useful for dating the infection. Recombinant proteins are highly advantageous for improving the diagnostic assay. The combination of several recombinant antigens with multiple immunodominant epitopes significantly increases the probability of detecting specific antibodies at different stages of the infection [4, 84]. Besides, avidity assays based on recombinant antigens have potential clinical usefulness for diagnosing the acute phase of *T. gondii* infection [26].

Many articles in this review assessed performance diagnosis or combined methods to evaluate performance improvement. The importance of diagnostic accuracy should be emphasized in order to conduct the correct treatment to avoid transplacental transmission and to prevent unnecessary and potentially toxic treatment or termination of pregnancy [5]. A combination of methods can also improve diagnostic accuracy [85]. *T*. *gondii* detection by DNA amplification or parasite isolation is complementary to serological tests. These methods are particularly important in AF to indicate fetal infection [86].

Our meta-analysis of diagnostic performance for DNA/parasite detection (PCR and bioassay) demonstrated a variation of sensitivity values. Although amniocentesis is a highly invasive method, amniotic fluid was the sample that presented the best values of PCR sensitivity. Likewise, detecting parasite burden in AF helps predict the severity of clinical symptoms in neonates congenitally infected [72]. The antenatal diagnosis of CT is the greatest advance in the cases of fetal infection, and the use of PCR analysis of AF is the most commonly used and accepted laboratory method for CT diagnosis during gestation [86]. Normally, negative PCR in AF indicates the absence of fetal infection, although it cannot be ruled out completely. However, a positive PCR result almost certainly indicates a congenital infection [14].

Variation in sensitivity can be associated with dissimilarity in the PCR methodologies, time of sample collection, time of maternal seroconversion, influence of treatment [19], and disparity in performance among laboratories. A considerable number of PCR results show the absence of *T. gondii* DNA amplification concomitant with CT, indicating low sensitivity [13]. In some cases, these results can be explained by the absence of optimal amniocentesis at the time of sample collection [23]. It can also be attributed to the inefficiency of parasite DNA extraction and amplification, mainly due to the low concentrations of tachyzoites in the AF collected [87].

Some studies in this review implemented comparisons of the sensitivities of PCR methodologies, including comparisons between target genes. By far, B1 is the most employed gene in PCR followed by REP529. There is an important discrepancy in the literature concerning the best target gene for PCRs. Some studies in this review indicated that REP529 is more sensitive compared to B1 [18, 68]. Another study showed no discordance between these two targets [88]. These results drive the need for more studies comparing target genes.

The present study aimed to estimate all possible random effects for CT diagnosis data and compare them without applying alternative simplifications [89]. Random effect correlations of +1 or −1 found for bioassay indicated a hit/truncation on model parameters. There were no explicit convergence problems, yet a few studies and/or sparse data (e.g., indicating no/low heterogeneity in the specificity parameter) are possibly data problems. In this situation, the power of the model can be compromised [90] and the diagnostic parameters should be interpreted with caution.

Our study to review pertinent publications and assess diagnostic test accuracy performance for detecting *T. gondii* infection was defined by the limited number of available studies that meet the selection criteria. Insufficient reporting regarding population characteristics/recruitment and data about sample number, sensitivity, and specificity was an issue in many studies, with information often provided with little detail.

Our results also indicate the need for searching for new diagnosis methodologies, improving existing techniques, and providing proper training for professionals involved in the routine diagnosis of CT [91]. Moreover, since higher *T. gondii* concentrations in AF are correlated with clinical signs in neonates, quantitative PCR can be important to evaluate the prognosis of the fetal infection [92]. Another important methodology that was scarcely used in selected articles, but is of great relevance, is *T. gondii* isolation followed by genotyping. This technique allows the identification of nonclonal strains. Dubey and coworkers [93] identified 58 different *T. gondii* genotypes circulating in Brazil. Studies identified a new *T. gondii* strain in southern Brazil that was strongly related to the toxoplasmosis outbreak in 2018 [94]. Such studies allow the surveillance of new circulating genotypes that are usually related to more severe forms of toxoplasmosis.

Variation in performance in diagnostics also drives the need for new diagnostic approaches, and few studies have focused on this aim. There is a great potential for using biomolecules present in AF for complementary diagnosis. Using complementary biomarkers, such as immune response mediators, could help endorse and increase the reliability of diagnosis in AF. Previous studies suggested the ability to use cytokines, such as TGF-*β* in AF, as biomarkers to predict acute *T. gondii* infection [95]. Another possibility is the use of AF cellularity as a potential biomarker of congenital infection. Studies using AF from the second and third trimester of pregnancy highlighted the cell dynamics in this compartment [96]. Inflammation, whether associated or not with infection, causes an increase in the number of immune cells.

Some studies in this review collected samples at birth, including AF, placenta, and blood. Samples collected at the time of delivery are especially significant in the absence of prenatal follow-up, making it possible to anticipate diagnosis and treatment for newborns. *T. gondii* isolation from placenta is a useful tool to study CT and is an easily available sample. Placental analysis can be important to diagnose infection when AF is either not positive or not analyzed. Besides, placental samples can be useful for isolation and genotyping of the parasite, especially in outbreaks [97]. However, it is important to highlight that maternal treatment can influence the efficacy of placental analysis since *T. gondii* was less frequently isolated in the placenta of treated women [62, 64, 66].

Postnatal follow-up, based on after-birth samples, remains necessary in the first year of life to fully exclude the infection when PCR or serological results were negative [29, 86]. Diagnosis based on cell immunity has been increasingly used as a complementary diagnostic to monitoring infants [98, 99]. This potential methodology should also be explored for maternal samples during prenatal follow-up. Alternative biological samples, such as colostrum from puerperal women [69] and saliva [44, 100], also provide interesting data on humoral immunity and promising results for diagnosing toxoplasmosis using noninvasive sample collection. Searching for IgG and IgG-subclasses produced by newborns compared with maternal antibody responses [32] can also be promising for CT diagnosis.

Preventive and diagnostic measures for pregnant women vary between countries. Although prenatal diagnosis of CT is available, there is no international framework for monitoring the disease, and it is a neglected disease in most countries [1]. The absence or incomplete prenatal screening and treatment have been identified as an important risk factor for CT [87]. Thus, the screening and prevention measures against toxoplasmosis should be made mandatory for pregnant women attending the antenatal clinic [4].

## 5. Conclusion

In conclusion, this review points out that the assays employed in the research are basically the same traditional approaches available for clinical purposes. These assays showed important variations in diagnostic performance that can result in undiagnosed CT. These results challenge us to search for new generations of diagnostic tools and improve existing techniques, together with efforts towards increasing the feasibility of laboratory testing.

## Figures and Tables

**Table 1:** Major goals of selected articles.

Samples in the major goal^†^				Analysis of performance	Diagnosis improvement	Comparisons	To distinguish acute and chronic phase of infection	Others	Year	Ref.
AF				To evaluate Se/Sp of a prenatal AF using PCR	—	To compare Se of PCR in AF according to intervals between amniocentesis and infection. To compare epidemiological parameters in children with positive and negative PCR results	—	To evaluate time of treatment and gestational age on CT diagnosis	2001	[5]
AF				—	To develop a duplex real-time PCR based on fluorescence resonance energy transfer to quantify parasite load and to determine assay sensitivity	To compare routine PCR and the lightcycler PCR	—	To correlate parasite load to ultrasonographic abnormalities. To correlate parasite load to the gestational age at the time of maternal infection	2001	[6]
AF				To evaluate Se/Sp of PCR and bioassay on AF	To combine PCR with mice inoculation to improve sensitivity	To compare Se/Sp between PCR and mouse inoculation. To compare prenatal and postnatal diagnosis	—	—	2002	[7]
AF				—	—	To compare *T. gondii* detection limit of 4 PCR methods in AF (conventional PCR, fluorescent PCR, real-time qPCR with SYBRGreen or with fluorescence energy transfer hybridization probe)	—	—	2006	[8]
AF				—	—	To compare different PCR methods (primers for B1, rDNA, and AF146527)	—	—	2009	[9]
AF				—	—	To compare 3 PCR assays for *T. gondii* detection in AF (commercial nested PCR and two laboratory-developed PCRs: conventional and real time)	—	—	2012	[10]
AF				To evaluate performance (Se, Sp, PPV, NPV, PLR, NLR and EF) of PCR in AF	—	To compare performance of 4 PCR methods for CT diagnosis in AF: nested multiplex, conventional, and real time	—	—	2013	[11]
AF				—		To compare 3 PCR assays used for *T. gondi*i infection diagnosis: P30-PCR, B1-PCR, and RE-PCR	—	To characterize the infecting *T. gondii* strains from the clinical specimen using B1 multicopy gene as target	2013	[12]
AF				To evaluate Se, Sp, PPV, NPV of PCR in AF to detect *T. gondii* infection	—	To compare Se of PCR performed at second or third trimester amniocentesis	—	To evaluate influence of treatment and timing of amniocenteses in PCR Se. To evaluate the outcome in infants with CT diagnosed by amniocenteses	2015	[13]
AF	BL			To evaluate Se/Sp of different diagnosis methods in AF and P-CB	To combine two diagnosis methods to improve diagnosis	To compare Se/Sp of different diagnosis methods	—	—	2002	[14]
AF	BL			—	To combine PCR with IgG avidity to improve CT diagnosis	—	—	—	2011	[15]
AF	BL			—	—	—	—	To characterize *T. gondii* present in AF and M-PB by genotyping	2012	[16]
AF	BL			—	—	—	—	To characterize atypical cases of *T. gondii* seroconversion (without IgM detection or with transient IgM levels) based on serology and PCR in AF	2013	[17]
AF	BL			To determine Se/Sp of diverse diagnosis methods and samples (AF, CB, and PB)	—	To compare prenatal and birth samples. To compare different PCR methodologies and different samples	—	—	2013	[18]
AF	BL			To verify the accuracy of the IgG avidity index to diagnose recent *T. gondii* infection	—	To compare IgG avidity index with PCR for *T. gondii* detection results in AF	—	To determine a cut-off value of IgG avidity to predict *T. gondii* DNA in AF	2015	[19]
AF	BL			To evaluate Se/Sp of PCR on CT diagnosis in AF obtained at birth	—	To compare Se in AF from patients with negative and positive CT diagnosis. To compare postnatal serology and at birth AF regarding early diagnosis ability	—	—	2015	[20]
AF	BL			—	To combine PCR and avidity IgG in order to improve performance diagnosis	To compare conventional ELISA and IgG avidity with PCR (genes B1 and P30) in BL and AF samples for early CT	To associate data of IgG anti-*T. gondii* titers, avidity index, and PCR to diagnose acute toxoplasmosis	—	2017	[21]
AF	BL			—	—	Comparison of gestational age, parasite load, and positive IgM between symptomatic and asymptomatic groups	—	To correlate *T. gondii* load, gestational age of maternal infection, and IgM at birth to the signs and severity of CT	2017	[22]
AF	BL			—	—	To compare PCR results for *T. gondii* in AF to the follow-up screen at birth	—	To describe *T. gondii* DNA detection in AF. To date maternal *T. gondii* infection	2018	[23]
	BL			—	—	To compare results obtained in the IgG avidity test with those obtained in the IgM ELISA and AC/HS tests	To evaluate the usefulness of testing for IgG avidity to exclude acute infection	—	2001	[24]
	BL			—	—	Comparison between IgM ELISA and VIDAS IgG avidity. Comparison between VIDAS IgG avidity test and TSp results (IgG, IgA, IgM, and IgE)	To differentiate recently acquired from distant infection using *T. gondii* IgG avidity (VIDAS kit) and TSp (IgG, IgA, IgM, and IgE)	—	2002	[25]
	BL			—	—	Comparison between individual recombinant antigens, its homogeneous mixture, and whole-cell toxoplasma antigen to determine IgG avidity	To discriminate between acute and latent phases of *T. gondii* infection by using recombinant antigens for avidity assay	—	2003	[26]
	BL			To evaluate Se/Sp for different AI thresholds	To define a new threshold to improve performance of avidity index for diagnosing chronic infection	—	To correlate time of infection with the avidity index in order to date infection	To evaluate if time and type of treatment influences the avidity index	2004	[27]
	BL			—	—	To compare IgM/IgG ELISA with PCR results performed in blood sample	—	To evaluate PCR utility to detect recent *T. gondii* infection	2004	[28]
	BL			—	—	—	To evaluate the ability of IgG-avidity to predict the risk of mother-to-child transmission of *T. gondii* in pregnant women IgG+/IgM+	—	2006	[29]
	BL			To evaluate Se, Sp, PPV, NPV of two commercial kits for acute toxoplasmosis designed to detect IgA and IgG avidity	To combine IgA and IgM tests to improve CT diagnosis	—	—	—	2007	[30]
	BL			—	—	To compare VIDAS avidity test and nested-PCR assay results to confirm ongoing or recent *T. gondii* infection in the selected group of pregnant women	To detect an ongoing or recent *T. gondii* infection in pregnant during the first 16 GW by using VIDAS *T. gondii* specific IgG-avidity test and nested PCR	—	2007	[31]
	BL			—	To improve CT diagnosis by comparison mother and child antibody subclasses (IgG1, IgG2, IgG3, and IgG4)	—	—	—	2008	[32]
	BL			To evaluate Se/Sp of IgG and IgM ELISA tests with rGRA6	—	The comparisons between ELISA GRA6 with ELISA VIDAS and ELISA euroimmun	To investigated rGRA6 potential to differentiate recently acquired infections to those acquired in the distant past	—	2008	[33]
	BL			To evaluate Se/Sp of IgM detection in filter paper-embedded blood	To verify if filter paper-embedded blood can be used for IgM detection. To determine IgG titer and IgG avidity in M-PB embedded in a filter paper	—	—	—	2009	[34]
	BL			To analyze Se/Sp of the ARCHITECT toxo IgG, IgM, and IgG avidity	—	To compare IgG and/or IgM results by ARCHITECT and 2 commercial techniques (AxSYM and VIDAS)	—	—	2009	[35]
	BL			To evaluate the ROC curve analysis of vitros IgM assay values for potential discrimination of acute and chronic infections	—	To compare the vitros IgG and IgM assays to the Sabin–Feldman dye test. To compare vitros Tg IgM to IgM ISAGA	To evaluate a screening serological method to identify chronic and acute Tg infection	—	2009	[36]
	BL			To evaluate Se, Sp, PPV, NPV of MEIA, ELFA, IFAT, and ELISA (IgM e IgA) for CT diagnosis	—	—	—	—	2009	[37]
	BL			To determine Se, Sp, PPV, NPV of combined elecsys IgG and IgM system	—	To compare the new Roche Elecsys *T. gondii* IgG and IgM immunoassay with Sabin–Feldman dye test and immunosorbent agglutination assay-IgM	To discriminate acute and chronic infection using ROC analysis for Elecsys IgM values	To determine the best cut-off using ROC analysis	2010	[38]
	BL			To evaluate performance of IgG avidity test based on recombinant GRA6 antigen to differentiate recently acquired and distant *T. gondii* infections	To develop an IgG avidity test based on recombinant GRA6 antigen	To compare ELISA to the euroimmun IgG avidity ELISA for exclusion of a recent *Toxoplasma* infection that occurred less than 4 months before	To evaluate IgG avidity based on rGRA6 assay ability to differentiate recently acquired and distant *T. gondii* infections in pregnant women	To determine the best parameters affecting the level of dissociation of antigen-antibody complex	2010	[39]
	BL			—	—	To compare immunoenzymatic, chemiluminescence, and indirect immunofluorescence assay with immunoblot analysis	To assess the immunoassays' abilities to diagnose seroconversion at its earliest stages	—	2011	[40]
	BL			—	—	—	To evaluate IgG avidity for TC diagnosis in early pregnancy	—	2011	[41]
	BL			—	To improve CT diagnosis by comparing mother and child antibody that target high-molecular-mass *T. gondii* antigens	—	—	To identify the best immunoblot bands of *T. gondii* antigens able to differentiate mother and child infection	2012	[42]
	BL			—	To identify potential *T. gondii* immunogens using pregnant sera and applying immunoproteomics assays	—	—	—	2012	[43]
	BL			To evaluate performance of LFIA for rapid screening of anti-*T. gondii* antibody in serum and saliva samples from pregnant women	Development of SAG2-LFIA, ROP2-LFIA, and SAG2 + ROP2-LFIA for rapid screening of anti-*T. gondii* ab in pregnant serum and saliva	To compare LFIA to commercial ELISA	Screening *T. gondii* IgM, IgG, and IgA avidity by SAG2 + ROP2-LFIA in order to detect recent or acute *T. gondii* infection	—	2012	[44]
	BL			—	—	The comparisons between PCR targeting the B1 gene and ELISA (IgG and IgM results) assays	—	—	2012	[45]
	BL			—	To determine the impact of additional maternal and/or N-CB serology on improving prenatal screening for CT	Comparison between prenatal and postnatal (M-PB and N-CB) serology	—	—	2013	[46]
	BL			To evaluate performance of IgG/IgM ELISA based on rMEP	To develop IgG/IgM ELISA based on rMEP	To compare rMEP-ELISA to commercial ELISA kits	To differentiate acute from chronic infection using ELISA based on a rMEP	—	2013	[47]
	BL			—	—	To compare EIA-IgG and FAT techniques in order to analyze equivocal or discordant results in routine IgG tests	—	To assess the usefulness of the WB as a confirmatory test to solve discordant results between EIA-IgG and FAT techniques	2013	[48]
	BL			To determine Se, Sp, PPV, NPV of VIDAS, architect and liaison systems for diagnosing *T. gondii* IgM and IgG	—	To compare anti-*T. gondii* IgG, IgM, and IgG avidity measurements obtained with three automated systems: VIDAS, architect, and liaison systems	—	To correlate anti-*T. gondii* IgG avidity measurements between VIDAS and architect and also between VIDAS and liaison systems	2013	[49]
	BL			—	—	To compare homemade WB with the commercial LDBIO II	—	To select the more valuable bands in a homemade WB that can be used as a confirmatory technique for discordant or equivocal results in EIA and FAT	2014	[50]
	BL			To determine Se, Sp, PPV, NPV of avidity assay	—	To compare IgG avidity results with the IgM and IgG ELISA test in a single serum sample	To discriminate acute and chronic infection in a single serum sample using IgG avidity assay	—	2014	[51]
	BL			To evaluate IgM and IgG ELISA Se/Sp based on PCR results	—	To compare immunological methods (ELISA IgM, IgG, and IgG avidity) to PCR based on 529 bp *T. gondii* DNA fragment	To investigate ELISA IgM and IgG-avidity and PCR results for detection of past or recent toxoplasmosis according to PCR results	—	2014	[52]
	BL			To determine performance of CML, indirect ELISA-rROP2 and IFI for the detection of IgG anti-*T. gondii*	—	To compare the performance of ELISA-rROP2 to CML and IFI for detection of IgG anti-*T. gondii.* Comparisons between IgG anti-*T. gondii* levels obtained from different pregnant groups using ELISA-rROP2 assay	—	—	2015	[53]
	BL			—	—	To compare VIDAS and architect avidity to determine the best method for estimating infection date	To estimate the date of infection	—	2016	[54]
	BL			To evaluate Se, Sp, PPV, NPV, accuracy of IgM IFAT in predicting recent infection according to the GW	—	—	To diagnose acute or chronic *T. gondii* infection using IgM IFAT	—	2016	[55]
	BL			To analyze AUC, Se, Sp of rP35a and rP22a to discriminate samples from not infected, typical-chronic, presumably acute, and recently chronic infections	Using bioinformatics tools to predict the highest epitopes density regions in P35 and P22 and expressed them for obtaining soluble proteins	To compare rP35a and rP22a performance to discriminate not infected, typical-chronic, presumably acute, and recently chronic infections	To assess the ability of both P35 and P22 antigens to differentiate *T. gondii* acute and chronic infection stages using indirect and avidity ELISA	—	2017	[56]
	BL			—	—	To compare IgM ELISA and IgG avidity in pregnant women during the first trimester pregnancy	To determine the performance of the IgG avidity test in detecting anti-*T. gondii* antibodies in pregnant women (IgG+/IgM+)	—	2017	[57]
	BL			—	—	To compare WB (LDBIO II) and automated assays (TGS TA, architect, vidasII, liaisonII, platelia, and Elecsys) concerning ability to detect early *T. gondii* seroconversion	—	To evaluated time required for anti *T. gondii* IgG detection by WB and 6 automated assays	2017	[58]
	BL			To determine Se, Sp of IIF, ELISA and IgG avidity tests	—	To compare ELISA, IIF and IgG avidity for diagnosing acute toxoplasmosis using a single serum sample	To discriminate acute and chronic infection using IgG avidity test	To evaluate the frequency of IIF and ELISA positivity using different serum dilutions	2017	[59]
	BL			To determine Se, Sp, PPV, NPV of IA assay based on latex particles	To develop IA assay based on LPC with *T. gondii* protein (P22Ag) to evaluate its ability of discrimination infected (chronic and acute) and noninfected control	To compare characteristics of LPC coupled with P22Ag and *T. gondii* homogenate. To compare the performance of LPC made of different compositions	To ruling out acute toxoplasmosis in pregnant women using IA based on LPC	—	2017	[60]
	BL			To evaluate the accuracy, Se, Sp, PPV, NPV of IgA in diagnosis of acute toxoplasmosis in pregnant women	—	To analyze IgA and IgM antibody positivity rates compared to AC/HS and IgG avidity results in pregnant women	To evaluate the usefulness of IgA in diagnosis of acute toxoplasmosis in pregnant women	—	2019	[61]
	BL	PL		To evaluate Se/Sp of methods (PCR, mouse inoculation) in PL and (ISAGA IgM, WB) in N-PB	To combine methods for improving Se diagnosis (PCR + bioassay in PL) or (ISAGA + WB in N-PB) or (PCR + bioassay in PL + ISAGA + WB in N-PB)	—	—	To establish the relationship between maternal treatment and *T. gondii* detection in PL. To analyze *T. gondii* strains isolated in PL tissue	2007	[62]
	BL	PL		To evaluate Se, Sp, PPV, NPV of PL for CT diagnosis using PCR and mouse inoculation	To combine mouse inoculation and PCR to improve sensitivity	—	—	To determine if time of maternal treatment or maternal seroconversion is related with PL Se	2010	[63]
AF	BL	PL		To evaluate Se, Sp, PPV, NPV of neonatal diagnosis: *T. gondii* isolation (PL and CB) and immunological tests (IgA and IgM). To evaluate Se of prenatal diagnosis	To combine prenatal and postnatal diagnosis for improving Se	To compare Se of different diagnosis methods	—	To evaluate treatment effect on diagnosis	2001	[64]
AF	BL	PL		—	—	Comparison between two PCR methods for detecting *T. gondii* in AF, BL, and tissues	—	—	2004	[65]
AF	BL	PL		To evaluate Se, Sp, PPV, NPV of methods (PCR, mouse inoculation) in AF and PL according to gestational age	—	To compare PCR and mouse inoculation in AF according to gestational age	—	—	2009	[66]
AF	BL	PL		To determine predictive values of molecular diagnosis in AF, PL, and CB	—	—	—	To estimate CT risk based on molecular diagnosis	2012	[67]
AF	BL	PL		To determine Se/Sp of different diagnosis methods and samples (AF, PL CB, and PB)	—	To compare two PCR methods for *T. gondii* detection in AF, CB, and PB	—	—	2015	[68]
	BL		CL	—	To evaluate CL as an alternative biological sample for CT diagnosis	To compare CL and M-PB samples in the following parameters: IgM, IgG, and IgA levels; IgG avidity against *T. gondii* antigenic fractions	—	To evaluate the correlation of IgM, IgG, and IgA detection in CL and M-PB samples	2015	[69]

^†^AF: amniotic fluid; BL: blood; PL: placenta; CL: colostrum; GW: gestational week. Ab: antibodies; AC/HS: differential agglutination (of acetone (AC)-fixed versus that of formalin (HS)-fixed tachyzoites); AUC: areas under the curve; CML: chemiluminescence; CT: congenital toxoplasmosis; EF: efficiency; EIA: enzyme immune assay; EIA: enzyme immunoassay; ELFA: enzyme-linked fluorescent assay; ELISA-rROP2: enzyme-linked immunoassay using recombinant *T. gondii* rhoptry 2; FAT: fluorescent antibody test; IA: immunoagglutination; IF: immunofluorescence test; IFAT: indirect‐fluorescent antibody test; IFI: indirect immunofluorescence; ISAGA: immunosorbent agglutination assay; LFIA: lateral flow immunoassay; LPC: latex-protein complexes; MEIA: microparticle enzyme immunoassay; M-PB: maternal peripherical blood; N-CB: neonatal cord blood; NLR: negative likelihood ratio; N-PB: neonatal peripherical blood; NPV: negative predictive value; P-CB: prenatal cord blood; PLR: positive likelihood ratio; PPV: positive predictive value; rGRA6: recombinant dense granule protein 6; rMEP: recombinant multiepitope fusion peptide; rP22a: recombinant soluble protein 22; rP35a: recombinant soluble protein 35; rtPCR: real-time PCR; Se: sensitivity; Sp: specificity; TSp: toxoplasma serological profile; WB: western blot.

**Table 2:** Diagnosis methods in amniotic fluid samples.

Diagnosis methods in amniotic fluid samples							
Time of sample collection				Target	Assay employed for diagnosis		Ref.
Gestational weeks			At birth	Parasite and/or DNA	PCR	Bioassay	
1 to 13	14 to 26	27 to 41				Mouse inoculation	
—	After 18 GW		—	Yes	B1 gene	(dnr)	[5]
[dnr]			—	Yes	B1 gene (nested-PCR)	Follow-up of mice serology and tissue cyst analysis	[7]
—	18–22	—	—	Yes	B1 gene	(dnr)	[14]
[dnr]			Yes	Yes	529-bp and B1 genes (real-time PCR)	(dnr)	[18]
—	22–33		—	Yes	[dnr]	Swiss Webster	[64]
—	18–39		Yes	Yes	B1 gene and RE sequence (conventional PCR-ELISA and real-time PCR)	(dnr)	[66]
[dnr]			—	Yes	B1 gene	(dnr)	[67]
—	After16 GW		—	Yes	[dnr]	(dnr)	[17]
—	15–34		—	Yes	B1 gene (conventional, fluorescent, and real-time qPCR with SYBR green or with fluorescence energy transfer hybridization probe)	—	[8]
—	14–25	—	—	Yes	AF146527 and B1 genes	—	[9]
[dnr]			—	Yes	Rep529 gene (commercial nested-PCR, conventional PCR, and real-time PCR)	—	[10]
—	16–25	—	—	Yes	B1 gene (conventional PCR, nested-PCR, multiplex-nested-PCR, and real-time PCR)	—	[11]
[dnr]			—	Yes	RE sequence and B1 and P30 genes (qPCR). SNPs within B1 gene for *T. gondii* DNA genotyping	—	[12]
—	From 18		Yes	Yes	529-bp gene (real-time PCR)	—	[20]
—	18–41		—	Yes	B1 gene (conventional PCR)	—	[13]
—	16–25	—	—	Yes	B1 gene (conventional PCR and qPCR)	—	[22]
—	17–28		Yes	Yes	B1, cdk, BSR4, and SAG5E genes (nested- PCR)	—	[15]
[dnr]			—	Yes	TGR1E gene (conventional PCR)	—	[16]
—	16–28		Yes	Yes	B1, cdk, SAG5E, and BSR4 genes (multiplex-nested-PCR)	—	[19]
—	14–16	—	—	Yes	B1 gene (nested-PCR), P30 gene (conventional PCR-ELISA)	—	[21]
[dnr]			—	Yes	B1 and 529pb genes (real-time PCR)	—	[23]
[dnr]			—	Yes	B1 gene	—	[65]
[dnr]			—	Yes	REP529 and B1 genes (qPCR)	—	[68]
[dnr]			—	Yes	B1 gene (duplex real-time quantitative light-cycler PCR)	—	[6]

dnr: details not reported; ELISA: enzyme-linked immunosorbent assay; GW: gestational weeks, CT: congenital toxoplasmosis; PCR: polymerase chain reaction; qPCR: quantitative PCR; SNPs: single nucleotide polymorphism; SSP: sequence-specific primers' amplifications.

**Table 3:** Diagnosis methods in blood samples.

Time of sample^†^ collection				Target		Assay employed for diagnosis^‡^				
Gestational weeks			At or after birth	Antibodies against *T. gondii*	Parasite and/or DNA	Immunoassay	PCR	Bioassay	Others	Ref.
1 to 13	14 to 26	27 to 41						Mouse inoculation		
M-PB			M-PB, N-CB, N-PB (follow-up during the first year of life)	IgM, IgG	—	ISAGA for IgM; SFDT for IgG	—	—	—	[46]
	—		N-CB	—	Yes	—	529-bp and B1 genes (qPCR)	—	—	[68]
M-PB	M-PB, P-CB		M-PB, N-CB, N-PB (follow-up during the 1st year of life)	IgM, IgG, IgA	Yes	[dnr] (M-PB); ISAGA and IMX toxo for IgM (P-CB, N-CB, N-PB); IFAT AND FEIA for IgG (N-CB, N-PB); ICT for IgA (P-CB, N-CB, N-PB); laser immunonephelometry for total IgG, IgM, and IgA (N-PB)	—	Swiss Webster female mice (P-CB and N-CB)	Laser immunonephelometry for C4 complement and orosomucoid (N-PB)	[64]
M-PB			M-PB, N-CB, N-PB (follow-up during the first year of life)	IgM, IgG, IgA, IgG avidity	Yes	IFAT, MEIA, and ISAGA for IgM (M-PB, N-PB); DA, MEIA, and IFAT for IgG (M-PB, N-PB); (dnr) for IgA (N-PB)	B1 gene (N-CB)	(dnr) (N-CB)	—	[67]
	—		N-CB, N-PB	IgM, IgA	—	ISAGA for IgM and ELISA for IgA	—	—	—	[66]
	—		M-PB, N-CB, N-PB (follow-up during the first year of life)	IgM, IgG	—	ISAGA, ELISA, and IFAT for IgM; WB for IgG and IgM	—	—	—	[62]
	—		M-PB	IgM, IgG, IgA, IgG avidity	—	ELISA for IgM, IgG, and IgA; WB for IgM, IgG, IgA, and IgG avidity	—	—	—	[69]
	—		M-PB, N-CB, N-PB (follow-up during the first year of life)	IgM, IgG	—	WB for IgM and IgG (M-PB, N-CB, N-PB); ISAGA for IgM and IgA (N-PB); immunoenzymatic method (VIDAS toxo IgGII, VIDAS toxo IgG, IgG enzygnost toxoplasmosis) for IgG (N-PB)	—	—	—	[42]
M-PB				IgM, IgG, IgA, IgG avidity	—	Architect for IgM; architect and VIDAS for IgG; platelia for IgA; architect and VIDAS for IgG avidity	—	—	—	[54]
M-PB			N-PB	IgG1, IgG2, IgG3, IgG4	—	ELISA	—	—	—	[32]
	—		N-CB, N-PB (follow-up during the first year of life)		Yes	—	529-bp and B1 genes (qPCR)	—	—	[18]
	P-CB		—	IgM, IgG, IgA	—	ISAGA for IgM; ELISA for IgG and IgA	—	—		[14]
M-PB			N-CB	IgM, IgG avidity	Yes	Plateria for IgM (N-CB); enzygnost for IgG avidity (M-PB)	B1, cdk, BSR4, and SAG5E genes (nested-PCR)	—	—	[15]
M-PB			—	IgM, IgG, IgG avidity	—	ELISA for IgM and IgG; platelia for IgG avidity	—	—	—	[57]
M-PB			N-CB	IgM, IgG	—	Enzygnost for IgG avidity (M-PB); plateria for IgM (N-CB)	—	—	—	[19]
M-PB			—	IgM, IgG	—	ECLIA (elecsys), WB, IFAT, and platelia for IgG; ECLIA (elecsys), platelia and ISAGA for IgM	—	—	—	[40]
M-PB			N-PB	IgM, IgG, IgG avidity	—	MEIA for IgG; ELFA, VIDAS for IgM; VIDAS and ELFA for IgG avidity	—	—	—	[29]
M-PB			N-PB	IgM, IgG, IgG avidity	—	FEIA for IgM (M-PB); ELISA for IgG and IgG avidity (M-PB, N-PB)	—	—	—	[34]
M-PB			—	IgM, IgG, IgA, IgE, IgG avidity	—	ELISA for IgM; SFDT, AC/HS for IgG; ELISA for IgA; ELISA and ISAGA for IgE; VIDAS for IgG avidity	—	—	—	[25]
M-PB			N-PB (follow-up during the first year of life)	IgM, IgG, IgG avidity	—	VIDAS, ELISA, WB for IgM (M-PB); VIDAS, enzygnost, and WB for IgG (M-PB); VIDAS for IgG avidity (M-PB); ISAGA and WB for IgM (N-PB); enzygnost and WB for IgG (N-PB)	—	—	—	[63]
M-PB			—	IgM, IgG, IgA, IgG avidity	—	VIDAS for IgM, IgG, and IgG avidity; platelia for IgA; recombinant protein enzyme immunoassay (GRA3, GRA7, MIC3, and SAG1) for IgG avidity	—	—	—	[26]
M-PB			M-PB, N-PB (follow-up during two year of life)	IgM, IgG, IgG avidity	—	Access, VIDAS and IFA for IgG; access, IFA and ISAGA for IgM; VIDAS for IgG avidity	—	—	—	[27]
M-PB			—	IgM, IgG, IgG avidity	—	MEIA, EIA, ISAGA, and architect for IgM; MEIA, EIA, IFI, SFDT, and architect for IgG; VIDAS and architect for IgG avidity	—	—	—	[35]
M-PB			—	IgM, IgG	—	ISAGA and vitros for IgM; SFDT and vitros for IgG	—	—	—	[36]
M-PB			—	IgM, IgG, IgA, IgG avidity	—	IF, platelia, and ISAGA for IgM; IF and platelia for IgG; platelia for IgA; platelia for IgG avidity	—	—	—	[30]
M-PB			—	IgG	—	ELISA and IFA for IgG; WB with sera against tachyzoites proteins	—	—	—	[43]
M-PB			—	IgM, IgG	Yes	ELISA for IgG and IgM	TGR1E gene, RFLP at SAG2 locus	—	—	[16]
M-PB			—	IgG	—	ELISA, SAG2-LFIA, ROP2-LFIA, and SAG2 + ROP2-LFIA	—	—	—	[44]
M-PB			—	IgM, IgG, IgA, IgG avidity	—	ELISA, IF, ISAGA, and rMEP-ELISA for IgM; ELISA, IF, and rMEP-ELISA for IgG; ISAGA for IgA; VIDAS for IgG avidity	—	—	—	[47]
M-PB			—	IgM, IgG	—	EIA (platelia-toxo IgM®) for IgM; EIA (platelia-toxo IgG®), FAT (toxospot IF®), and WB (LDBIO-toxo II IgG®) for IgG	—	—	—	[48]
M-PB			N-PB	IgM, IgG, IgG avidity	—	VIDAS, architect, liaison, IFAT, ISAGA for IgM, VIDAS, architect, liaison, IFAT, and SFDT for IgG; VIDAS, architect, and liaison IgG avidity	—	—	—	[49]
M-PB			—	IgM, IgG, IgG avidity	—	CML for IgM; IFI, CML, and ELISA-rROP2 for IgG; CML for IgG avidity	—	—	—	[53]
M-PB			—	IgM, IgG, IgG avidity	—	ELFA, IFAT for IgM; ELFA, and IFAT for IgG; ELISA for IgG avidity	—	—	—	[55]
M-PB			—	IgM, IgG	—	Architect, platelia, and VIDAS for IgM; architect, platelia, VIDAS, liaison toxo IgG II®, elecsys toxo IgG® TGS TA toxo IgG®, and WB (LDBIO-toxo II IgG®) for IgG	—	—	—	[58]
M-PB			—	IgM, IgG, IgA, IgG avidity	Yes	ELISA for IgM, IgG, IgA, and IgG avidity	B1 gene (nested PCR); P30 gene (PCR-ELISA)	—	—	[21]
M-PB			—	IgM, IgG	—	EIA (platelia-toxo IgM®) for IgM; EIA (platelia-toxo IgG®), FAT (ToxoSpot IF®), WB (LDBIO-toxo II IgG western blot®), and homemade WB with soluble tachyzoite antigens for IgG	—	—	—	[50]
M-PB			—	IgM, IgG, IgA, IgE IgG avidity	—	ELISA for IgM; SFDT and AC/HS for IgG; ELISA for IgA; ELISA and ISAGA for IgE; EIA for IgG avidity	—	—	—	[24]
M-PB			N-PB	IgM, IgG, IgG avidity	—	ISAGA for IgM; SFDT for IgG	—	—	—	[13]
M-PB			—	IgM, IgG, IgG avidity	Yes	Latex agglutination test (latex-toxo kit ELISA) for IgM; ELISA for IgG; ELISA for IgG avidity	529-bp	—	—	[52]
M-PB			—	IgM, IgG, IgG avidity	—	ELISA for IgM; IFAT and ELISA with rP35a or rP22a proteins for IgG; ELISA (VIDAS) and ELISA with rP35a or rP22a proteins for IgG avidity	—	—	—	[56]
M-PB			—	IgM, IgG, IgG avidity	—	ELISA for IgM; IFAT and ELISA with P22Ag protein for IgG; ELISA (VIDAS) and IA with LPC-P22Ag protein for IgG avidity	—	—	—	[60]
M-PB			—	IgM, IgG, IgG avidity	—	IF and ELISA for IgM; IF and ELISA for IgG, EIA for IgG avidity	—	—	—	[59]
M-PB			—	IgM, IgG, IgG avidity	—	ELISA (platelia) for IgM; ELISA (platelia) for IgG; platelia for IgG avidity	—	—	—	[51]
M-PB			—	IgM, IgG	—	ISAGA and ECLIA for IgM; SFDT and ECLIA for IgG	—	—	—	[38]
M-PB			—	IgM, IgG, IgG avidity	—	VIDAS, IF, ISAGA, and euroimmun ELISA for IgM; VIDAS, IF, euroimmun ELISA and ELISA with GRA6 protein for IgG for IgG; euroimmun ELISA and ELISA with GRA6 protein for IgG avidity	—	—	—	[39]
M-PB			—	IgM, IgG, IgA, IgG avidity	—	ELISA for IgM; SFDT and AC/HS for IgG; ELISA for IgA; VIDAS for IgG avidity	—	—	—	[61]
M-PB			—	IgM, IgG	—	VIDAS, IF, ISAGA, euroimmun ELISA, and ELISA with rGRA6 for IgM; VIDAS, IF, euroimmun ELISA, and ELISA with rGRA6 for IgG	—	—	—	[33]
M-PB			—	IgM, IgG	Yes	ELISA for IgM and IgG	B1 gene	—	—	[45]
M-PB			—	IgM, IgG, IgG avidity	Yes	VIDAS for IgM, IgG, and IgG avidity	B1 gene (nested PCR)	—	—	[31]
M-PB			—	IgM, IgG	Yes	ELISA for IgM and IgG	B1 gene (nested PCR)	—	—	[28]
M-PB			—		Yes	—	B1 gene	—	—	[65]
M-PB			N-PB (follow-up during two years of life)	IgM, IgG, IgG avidity	—	IF for IgM and IgG (M-PB); VIDAS for IgG avidity (M-PB); [dnr] for IgM and IgG (N-PB)	—	—	Blood count and liver enzymes (N-PB)	[22]
M-PB			M-PB, N-PB	IgM, IgG, IgG avidity	—	ELISA and WB for IgM (M-PB, N-PB); ISAGA for IgM (N-PB); ELISA and WB for IgG (M-PB, N-PB); ELISA for IgG avidity (M-PB)	—	—	—	[23]
M-PB			N-CB, N-PB (follow-up during two years of life)	IgM, IgG, IgA, IgG avidity	—	ELISA for IgM (M-PB); ELISA for IgG avidity (M-PB); [dnr] for IgM and IgG (N-CB); MEIA, ELFA and IFAT for IgM (N-PB), MEIA for IgG (N-PB); ELISA for IgA (N-PB)	—	—	—	[37]
M-PB			—	IgM, IgG, IgG avidity	—	ELISA for IgM and IgG; ELISA euroimmun for IgG avidity	—	—	—	[41]
M-PB			N-PB (follow-up during two years of life)	IgM, IgG, IgA, IgG avidity	—	VIDAS, vidia, AxSYM, architect, cobas, enzygnost, advia centaur, platelia, liaison, ISAGA, ICT, and IFAT for IgM (M-PB); VIDAS, vidia, AxSYM, architect, cobas, enzygnost, advia centaur, platelia, liaison, IFAT, toxo-screen, WB (LDBio toxo II IgG), HSDA, IA, and SFDT for IgG (M-PB); ISAGA, ICT, and platelia for IgA (M-PB); VIDAS, platelia, and architect for IgG avidity (M-PB); EIA, ISAGA, ICT, WB, and ELIFA for IgM and IgA (N-PB); EIA, ISAGA, and ICT for IgA (N-PB)	—	—	—	[17]

^†^M-PB: maternal peripherical blood; N-CB: neonatal cord blood; P-CB: prenatal cord blood; N-PB: neonatal peripherical blood. ^‡^AC/HS: differential agglutination (of acetone [AC]-fixed versus that of formalin [HS]-fixed tachyzoites); CIP-ELIFA: comparative immunological profile (CIP) method with revelation by enzyme-linked immunofiltration assay (ELIFA); CML: chemiluminescence; DA: direct agglutination assay; DNA: deoxyribonucleic acid; dnr: details not reported; ECLIA: electrochemiluminescence immunoassay; EIA: enzyme immunoassay; ELFA: enzyme-linked fluorescent assay; ELIFA: enzyme-linked immunofiltration assay; ELISA: enzyme-linked immunosorbent assay; FAT: fluorescent antibody test; FEIA: fluorometric enzyme immunoassay; GGT: gamma glutamyl transpeptidase; HSDA: high-sensitivity direct agglutination; IA: immunoagglutination; ICT: immunocapture method using a suspension of tachyzoites prepared in the laboratory; IF: immunofluorescence test; IFAT: indirect-fluorescent antibody test; IMX: Abbott IMx automated benchtop immunochemistry analyzer system (MEIA + fluorescence polarization immunoassay (FPIA)); ISAGA: immunosorbent agglutination assay; LDH: lactate dehydrogenase; LFIA: lateral flow immunoassay; LPC: latex-protein complexes; MEIA: microparticle enzyme immunoassay; PCR: polymerase chain reaction; q-PCR: quantitative polymerase chain reaction; rMEP: recombinant multi-epitope fusion peptide; SFDT: Sabin–Feldman dye test; VIDAS: immunodiagnostic assay system; WB: western blot; y-GT: y glutamyltranspeptidase.

**Table 4:** Diagnosis methods in postnatal samples.

Sample	Time of sample collection	Target		Assay employed for diagnosis			Ref.
	At or after birth	Antibodies against *T. gondii*	Parasite and DNA	Immunoassay	PCR	Bioassay	
						Mouse inoculation	
PL	Yes	—	Yes	—	REP529 and B1 genes (qPCR)	—	[68]
	Yes	—	Yes	—	B1 gene	—	[65]
	Yes	—	Yes	—	REP529 gene (real-time PCR)	Swiss IOPS female mice	[63]
	Yes	—	Yes	—	B1 gene	[dnr]	[62, 67]
	Yes	—	Yes	—	B1 gene and RE sequence (conventional PCR-ELISA and real-time PCR)	[dnr]	[66]
	Yes	—	Yes	—	—	Swiss Webster female mice	[64]

CL	Yes	IgM, IgG, IgA, and IgG avidity	—	ELISA (IgM, IgG, and IgA) and western blot (IgM, IgG, IgA, and IgG avidity)	—	—	[69]

PL: placenta; CL: colostrum; DNA: deoxyribonucleic acid; dnr: details not reported; PCR: polymerase chain reaction; qPCR: quantitative PCR; ELISA: enzyme-linked immunosorbent assay.

**Table 5:** Summary estimates of diagnostic accuracy of molecular and bioassay techniques for the diagnosis of *T. gondii* infection.

Parameter	PCR–AF			Bio–AF			PCR–PLA			Bio–PLA		
	Estimate	2.5% CI	97.5% CI	Estimate	2.5% CI	97.5% CI	Estimate	2.5% CI	97.5% CI	Estimate	2.5% CI	97.5% CI
Sensitivity	0.851	0.695	0.934	0.574	0.416	0.718	0.589	0.585	0.593	0.586	0.472	0.692
Specificity	0.997	0.972	1.000	0.993	0.936	0.999	0.963	0.963	0.964	0.995	0.979	0.999
Random effects correlation	−0.572			1.000			−0.365			−1.000		
Diagnostic odds ratio	2018.385	228.652	17816.960	189.944	13.453	2681.756	37.706	36.843	38.588	258.867	69.775	960.405
Likelihood ratio (LR+)	302.048	30.916	2950.945	81.498	7.388	899.084	16.085	15.815	16.360	107.661	28.853	401.718
Likelihood ratio (LR−)	0.150	0.069	0.325	0.429	0.295	0.623	0.427	0.422	0.431	0.416	0.318	0.544

PCR: polymerase chain reaction; AF: amniotic fluid; Bio: bioassay; PLA: placenta.

## Data Availability

The data used to support the findings of this systematic review are available in the References section of the article.

## Additional Points

*What is Already Known about This Topic?* (i) Advances in immunology, molecular biology, and bioinformatics have provided a new generation of diagnostic tools. (ii) CT diagnosis is essential for the treatment and clinical management of disease. (iii) New possibilities for diagnostic tools have been searched to improve the diagnosis of acquired toxoplasmosis. (iv) Pregnancy provides many potential samples to be used in CT diagnosis, presenting a wide array of new possibilities. *What Does This Study Add?* (i) Pregnancy samples have been poorly explored for searching the new generation of diagnostic tools. (ii) The assays used in the research to diagnose CT are basically the same traditional approaches available for clinical purposes. (iii) PCR and bioassay showed large variations in diagnostic performance. (iv) Improvement and searching for new forms of diagnosis are required for CT. (v) The search for immunological markers, the use of *T. gondii* recombinant antigens, and the identification of the genetic diversity of *T. gondii* strains are diagnostic possibilities to be explored using pregnancy samples.
